# Lactic acid fermentation using *Rhizopus* spp.: current insights and future prospects

**DOI:** 10.1186/s40643-025-00982-6

**Published:** 2025-12-03

**Authors:** Mst. Mahmoda Akter, Marium Akter Jim, Brandon Gilroyed

**Affiliations:** 1 School of Environmental Sciences, University of Guelph Ridgetown Campus, 120 Main Street East, Ridgetown, ON N0P 2C0 Canada; 2 Centre for Agricultural Renewable Energy and Sustainability (CARES), University of Guelph, Ridgetown Campus, Ridgetown, N0P 2C0 Canada; 3 Department of Environmental Science and Disaster Management, Noakhali Science and Technology University, Noakhali, 3814 Bangladesh

**Keywords:** Lactic acid, Fungal fermentation, *Rhizopus*, Bioprocess optimization, Renewable feedstocks, Polylactic acid (PLA), Downstream purification, Techno-economic analysis

## Abstract

Lactic acid (LA) is a versatile organic acid widely used in food, chemical, pharmaceutical, and cosmetics industries. Its demand has significantly increased due to its role in producing biodegradable and biocompatible polylactic acid (PLA) polymers. Fungal species from the *Rhizopus* genus offer several advantages over bacteria when producing lactic acid through fermentation of renewable substrates, including amylolytic capabilities, minimal nutrient requirements, and valuable fungal biomass as a by-product. This review highlights recent advancements in the metabolic and enzymatic pathways, fermentation substrates, modes, and methods utilized in LA production by *Rhizopus* species. It explores critical bioprocess parameters such as nutrient composition, pH, and fungal morphology, which are examined for their roles in optimizing production. Furthermore, developments in high cell-density fermentation and improved downstream processes for lactic acid recovery and purification are discussed. The challenges and opportunities for scaling up LA production from various substrates are critically analyzed, along with future strategies for improving fungal fermentation systems. Finally, the techno-economic feasibility of fungal-based LA production is also discussed.

**Graphical abstract:**

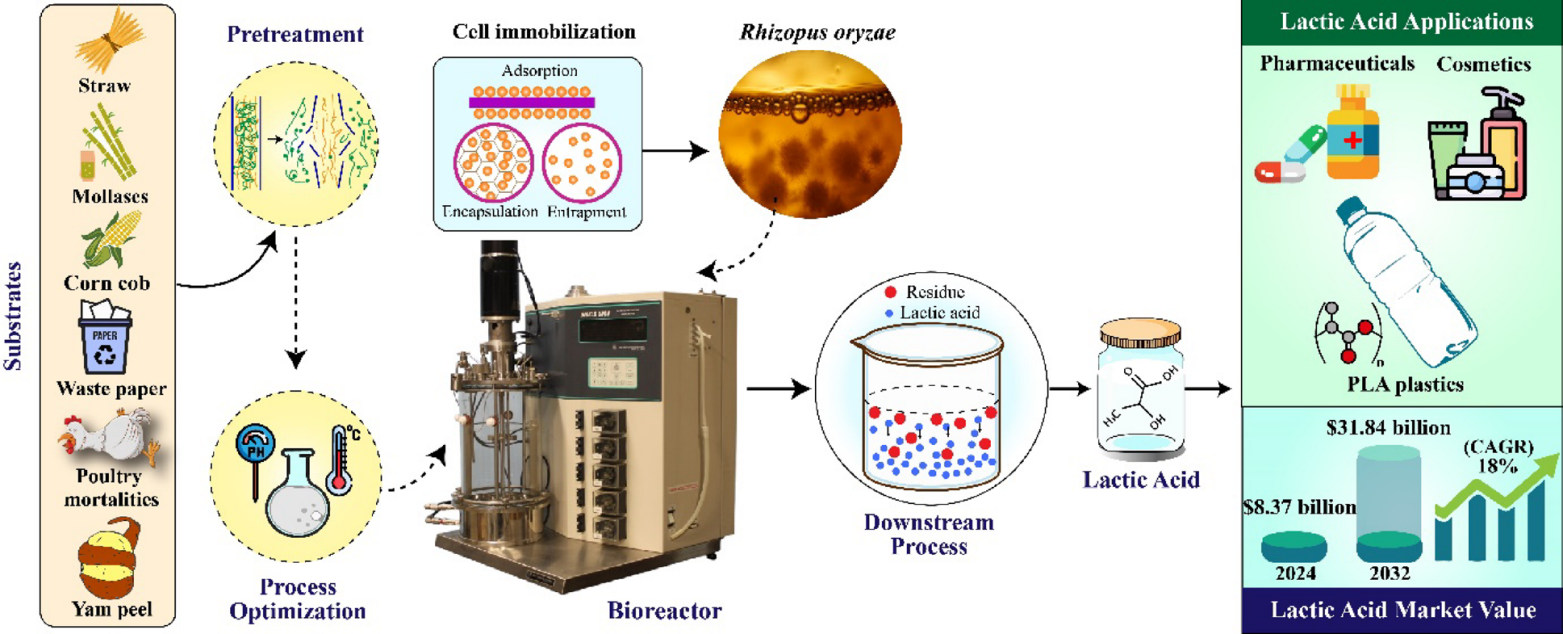

## Introduction

**Fig. 1:**
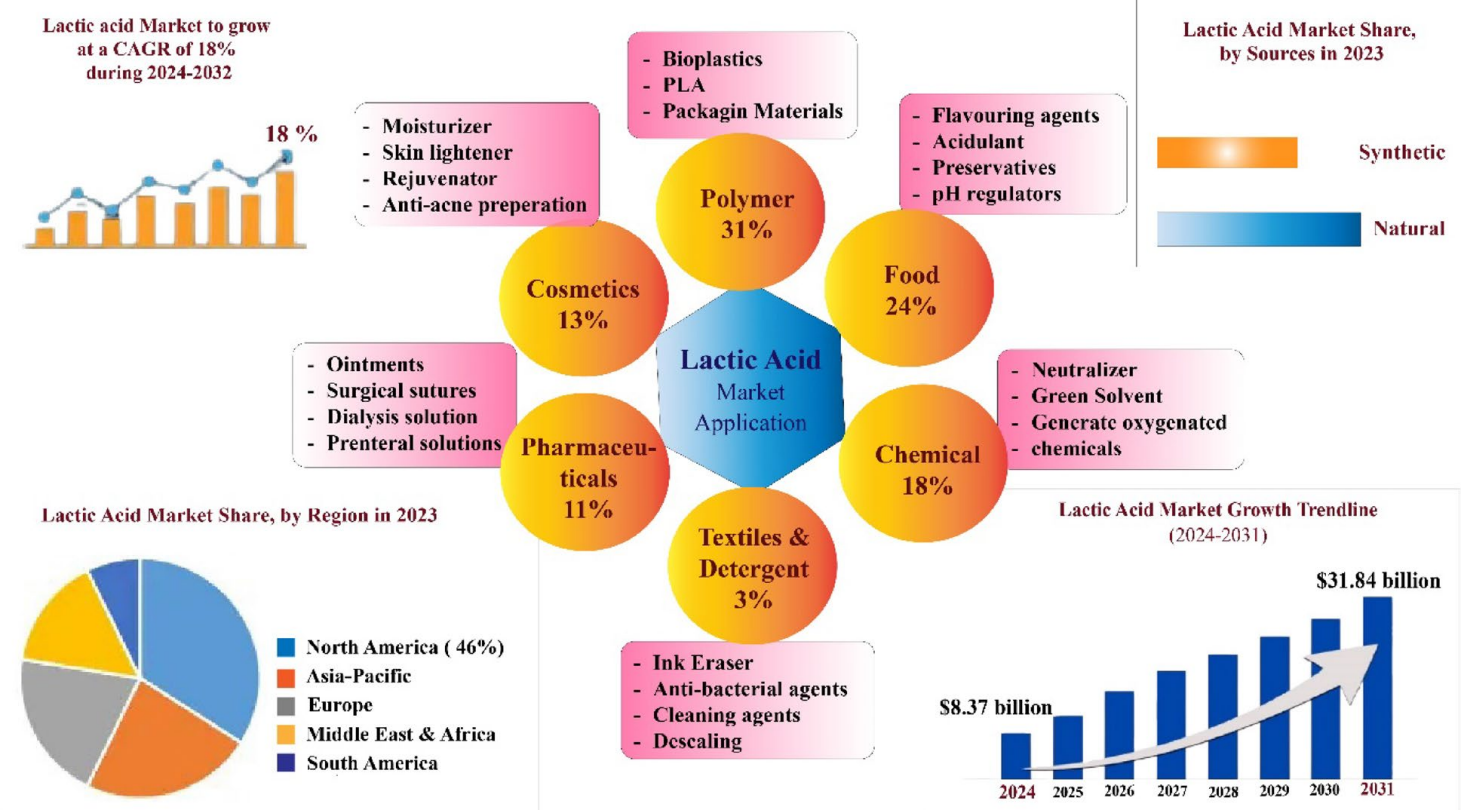
Lactic acid market and its diversified applications across various industries. Adopted from (Huang et al. 2023)

Traditionally plastics are derived from petroleum, but the rapid depletion of fossil fuels and growing environmental concerns have driven the development of sustainable alternatives. These approaches focus on producing recyclable and biodegradable plastics from renewable biomass feedstocks (Rodríguez-Torres et al. 2022; Göçeri̇ et al. 2021; Abdel-Rahman et al. 2013). Lactic acid (LA) (C_3_H_6_O_3_), in its optically pure L- and D-forms, serves as the precursor for biodegradable polylactic acid (PLA), a key component of bioplastics used in products such as packaging, films, foams, and fibers (Abedi and Hashemi 2020; Ahmad et al. 2020; Lawal et al. 2024). LA was first discovered by Carl Wilhelm Scheele in sour milk in 1780, and its industrial production through microbial fermentation was developed by the French scientist Frémy in 1881 (Vaidya et al. 2005). It is in high demand and widely used across food, pharmaceuticals, cosmetics, detergents, and dairy industries, and can be converted into other acids, esters, and bio-solvents (Juturu and Wu 2016; Garavand et al. 2018; Coelho et al. 2020; Rodríguez-Torres et al. 2022) (Fig. 1).

The global LA market was valued at USD 8.37 billion in 2024 and is projected to reach USD 31.84 billion by 2032, while the global PLA market is expected to grow from USD 1.59 billion in 2024 to USD 3.18 billion by 2028 (Fortune Business Insights 2025; The Business Research Company 2025) (Fig. 1). LA, typically sold as an 88% solution, price varies with feedstock, and demand is projected to reach 1,960.1 kilotons by 2025, driven mainly by medical and cosmetics sectors in Latin America and the Asia–Pacific (Abedi and Hashemi 2020; Zwiercheczewski De Oliveira et al. 2022; Ahmad et al. 2024; Pinlova et al. 2024) (Fig. 1). Despite rising demand, the high cost of LA and PLA production remains a challenge, with commercial viability dependent on reducing production costs below $0.80 per kg and its selling price must be reduced to half of the current market rate (Pal et al. 2009; Ilyas et al. 2022; Ojo and De Smidt 2023; Lawal et al. 2024).

Lactic acid is produced through two primary methods: chemical synthesis and microbial fermentation. Chemical synthesis yields a racemic mixture of D/L lactic acid, whereas microbial fermentation is preferred due to its advantages, including producing optically pure L(+) or D(−) LA, lower energy requirements, and the use of renewable substrates (Abdel-Rahman et al. 2011, 2013; Rawoof et al. 2021). The choice of carbon source significantly impacts production costs. Traditional feedstocks like glucose from starchy materials (e.g., corn, wheat, and potato starch) are widely used but compete with food supplies, raising concerns about sustainability (Saito et al. 2012; Akoetey and Morawicki 2018; Göçeri̇ et al. 2021). As a result, lignocellulosic biomass (e.g. corn cobs, wood) and sugarcane molasses have emerged as promising, cost-effective alternatives for lactic acid production, supported by advancements in pretreatment and fermentation technologies (Groff et al. 2022; Huang et al. 2023; Ojo and De Smidt 2023).

Lactic acid fermentation can be carried out by various microorganisms, including bacteria and fungi. Some common microorganisms used today include *Lactobacillus, Bacillus, Escherichia coli, Rhizopus oryzae, Aspergillus niger,* and *Saccharomyces cerevisiae*. Among them, lactic acid bacteria (LAB) are widely used in industrial-scale production due to their high growth rates and product yields (Huang et al. 2023). However, LAB fermentation can increase downstream costs since these bacteria require complex nutrients to synthesize B vitamins and amino acids. Among fungi, *Rhizopus oryzae* is particularly attractive for lactic acid production due to its ability to grow in nitrogen-limited environments, form filamentous or pellet morphologies that simplify downstream separation, and require minimal inorganic salts, which together reduce production costs (Fu et al. 2009; Meussen et al. 2012; Dörsam et al. 2017). The species was first reported in 1911 to produce LA via surface culture, and in 1936 an efficient submerged fermentation process was developed (Zhang et al. 2007a). Moreover, the fungal biomass from *Rhizopus* strains can serve as a by-product in biosorption processes for the purification of contaminated effluents or as an additive in animal feeds, offering advantages not commonly observed in other fungal systems (Krzyżaniak et al. 2013; Kumar et al. 2019b; Huang et al. 2023; Zhang et al. 2007b). Nevertheless, the recovery and purification of LA from fermentation broth remain significant challenges due to the complex media, which complicates separation and purification processes. The main cost drivers in LA production are feedstock and downstream processing (Pleissner et al. 2013; López-Gómez et al. 2020). While agricultural byproducts offer a sustainable feedstock alternative, they present difficulties during pretreatment, producing inhibitory compounds that reduce productivity. Additionally, residual sugars, impurities, and organic acids lower the optical purity of LA. These issues increase recovery costs and hinder the economic feasibility of the process.

This article reviews recent research on LA production by fungi, particularly *Rhizopus* species, emphasizing their advantages over bacterial fermentation. It discussed the key process components, renewable substrates, metabolic pathways, fermentation modes, and media used in fungal LA production. The impact of critical bioprocess parameters, such as nutrient composition, pH, and growth morphology, is analyzed. Additionally, the review highlights advance in high-cell-density fermentation, downstream processing, and evaluates the limitations, challenges, techno-economic feasibility, and future prospects of these methods.

## Fungal-based lactic acid fermentation

### Pros and cons of fungi vs bacteria

LA fermentation is pivotal in producing sustainable chemicals for industries such as food, pharmaceuticals, bioplastics, chemical and cosmetics industries. Microbial systems, including fungi and bacteria, have been extensively studied for their potential in this process. Each microorganism has unique advantages and disadvantages, which determine its suitability for specific applications.

#### Fungi in lactic acid fermentation

Fungi, particularly *Rhizopus* species, are well-known for their production of optically pure L-LA. Due to their ability to flourish in acidic environments, the need for stringent pH control and costly neutralizing agents is reduced (Taskin et al. 2012; Wu et al. 2017). Compared to LA-producing bacteria, *Rhizopus* strains offer several advantages, such as their amylolytic abilities, which enable the direct utilization of starchy biomasses without requiring prior saccharification (Jin et al. 2005). Additionally, these fungi have minimal nutrient requirements (Dörsam et al. 2017) and simplify downstream processing due to their filamentous or pellet growth, which allows easier separation from the fermentation broth compared to bacterial or yeast cells (Zhang et al. 2007b). The fungal biomass generated during fermentation also serves as a valuable byproduct. The morphology of fungal growth, including forms such as filamentous structures, mycelial mats, pellets, or clumps, significantly impacts broth rheology, oxygen transfer, and lactic acid yield. For industrial applications, small fungal pellets are preferred as they improve mass transfer and rheological properties, facilitating long-term operations through repeated batch fermentations (Meussen et al. 2012). While immobilization techniques have been explored for L-lactic acid production using *R. oryzae* (Shahri et al. 2020) these methods often face limitations due to the time required for cell entrapment on matrices and constraints on operational volume.

However, there are some challenges associated with using filamentous fungi for LA production. For example, in nitrogen-rich media, fungi tend to grow rapidly and produce chitin instead of LA. To address this, strategies such as using fungal pellets instead of spores, increasing carbon content, and reducing nitrogen sources have been suggested to improve LA yield (Liu et al. 2006). Additionally, there is limited information on direct LA production by other fungal species. In one case, *Aspergillus niger* was used in combination with *Lactobacillus* sp. for simultaneous saccharification and fermentation of artichoke to LA, where the fungus supplied the necessary enzymes for the breakdown of carbohydrate polymers into fermentable sugars (Ge et al. 2009). Another limitation of fungi is their slower growth rates compared to bacteria, which results in longer fermentation times (Wee et al. 2006; John et al. 2007). Furthermore, fungi generally require oxygen for growth, which increases the energy requirements of the fermentation process, making it more energy-intensive than bacterial fermentation.

#### Bacteria in lactic acid fermentation

Lactic acid is produced by various bacterial genera, either as a primary or secondary fermentation product. LAB specifically refers to those genera within the order *Lactobacillales*, which includes species such as *Lactobacillus, Pediococcus, Aerococcus, Carnobacterium, Enterococcus, Tetragenococcus, Vagococcus, Leuconostoc, Oenococcus, Weissella, Streptococcus*, and *Lactococcus* (Reddy et al. 2008; Abdel-Rahman et al. 2011). Among LAB, species of the genus *Lactobacillus* are particularly recognized for their rapid growth and effective conversion of glucose into lactic acid with minimal by-products, making them a preferred choice for industrial lactic acid production (Abdel-Rahman et al. 2013; Lee et al. 2015). Additionally, these bacteria can be genetically manipulated, allowing for the optimization of their substrate utilization and better control over the purity of the stereoisomers produced.

However, using bacterial systems for LA fermentation has its drawbacks. A major limitation is their sensitivity to acidic conditions, which necessitates constant pH control and the addition of neutralizing agents to prevent metabolic inhibition. Most LAB strains preferentially utilize simple sugars (e.g., glucose) as carbon sources, necessitating pretreatment of lignocellulosic biomass to release fermentable sugars—a step also required for many other microorganisms, which increases production costs (Huang et al. 2023; Ojo and De Smidt 2023). In contrast, engineered strains of *Escherichia coli* (a non-LAB) can also produce lactic acid by metabolizing both pentose and hexose sugars derived from untreated biomass, though wild-type *E. coli* typically requires genetic modifications and can grow on simpler media, such as Lysogeny broth (LB), which is less costly compared to the media needed for LAB (Okano et al. 2010). Furthermore, *E. coli* benefits from simpler plasmid transformation and gene knockout processes compared to LAB or *Bacillus* species. However, a significant challenge with *E. coli* is the inefficient carbon flux, leading to the production of a mixture of acids (including D-LA, acetic acid, succinic acid, formic acid) and ethanol, which diminishes the yield and purity of lactic acid (Klotz et al. 2016). Table 1 outlines the advantages and disadvantages of using fungi and bacteria for lactic acid (LA) fermentation.

**Table 1:** Comparison of advantages and limitations of fungi and bacteria in lactic acid fermentation

Aspect	Fungi (*Rhizopus oryzae*)	Bacteria (e.g., *Lactobacillus* spp. and *Bacillus coagulans*)
*Pros*		
Lactic acid (LA) yield	80–90% theoretical yield under optimized conditions, especially with glucose as a substrate	*Lactobacillus*: 90–95% of theoretical yield (anaerobic) *B. coagulans*: > 90% (L-LA exclusively, consumes pentoses/hexoses)
pH Tolerance	Tolerates pH 3.0–4.0 (low contamination risk)	*Lactobacillus*: Sensitive to pH < 4.5 *B. coagulans*: Thermotolerant (50–55 °C), pH-stable
Substrate Range	*R. oryzae* efficiently ferments complex substrates like lignocellulosic biomass and agricultural residues (Kupski et al., 2015)	*Lactobacillus*: Prefers glucose/lactose *B. coagulans*: Utilizes diverse sugars (C5/C6)
Product Purity	Predominantly L( +)-LA; minor by-products (ethanol/glycerol)	*Lactobacillus*: High-purity L( +)-LA *B. coagulans*: Optical purity (> 99% L-LA)
Fermentation Type	Solid-state (SSF) for lignocellulosic	*Lactobacillus*: Submerged (SmF); *B. coagulans* suits high-temperature SmF
Metabolic Pathways	*R. oryzae* produces LA via the fermentative glycolytic pathway (Embden-Meyerhof-Parnas, EMP pathway)	*Lactobacillus*: Homofermentative EMP; *B. coagulans*: Similar, with thermostable enzymes
*Cons*		
Growth Rate	Slow (doubling time: 5–10 h)	Fast (*Lactobacillus*: 20–60 min; *B. coagulans*: ~ 1 h)
Nutrient Requirements	Requires standard fungal media (peptone, yeast extract)	Simple (*Lactobacillus*: glucose/lactose; *B. coagulans*: minimal media)
Fermentation Conditions	Requires precise control of temperature (optimal at 30–35 °C) and aeration (1–2 vvm for aerobic fermentation)	*Lactobacillus*: Needs pH control; *B. coagulans*: Tolerates harsh conditions
Production Cost	Higher costs due to slow growth, complex nutrient needs, and aeration requirements	Lower (*Lactobacillus*: rapid growth; *B. coagulans*: reduced sterilization costs)
Genetic Manipulation	Difficult to genetically modify due to multinucleated structure and limited genetic tools	Easier (*Lactobacillus*/*B. coagulans*: established tools)
Contamination Risk	Low (acidic pH tolerance)	Higher (*Lactobacillus*: neutral pH vulnerability; *B. coagulans*: reduced risk at high temps)

### Fungal mechanisms of lactic acid fermentation

**Fig. 2:**
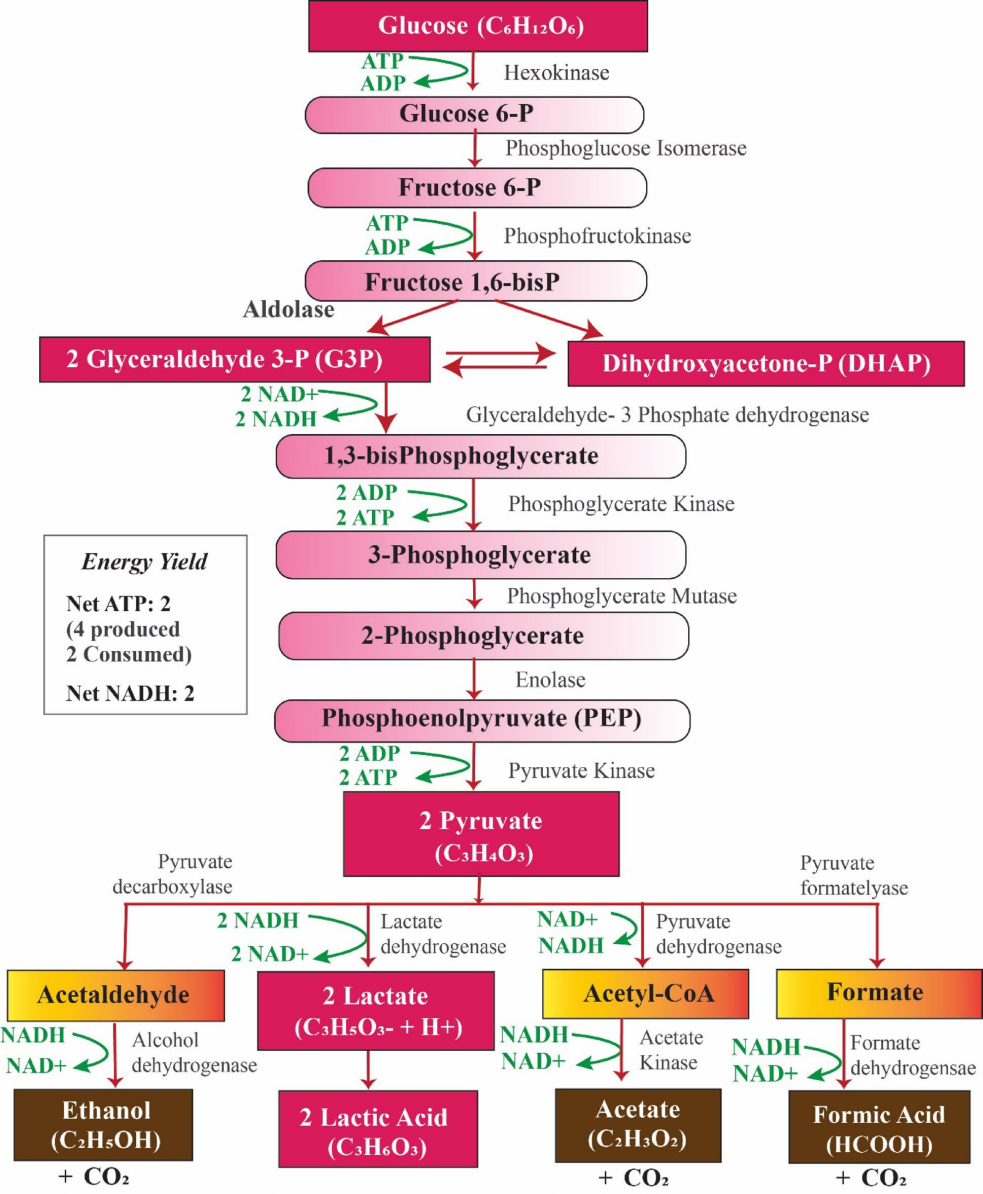
Metabolic pathway of lactic acid fermentation in *Rhizopus oryzae* under anaerobic conditions

Fungal metabolism, especially in LA production, occurs through anaerobic processes. A diagram that illustrates the key reactions involved in producing LA fermentation by using fungi from glucose is presented in Fig. 2 (Meussen et al. 2012). This pathway (Embden-Meyerhof Pathway) details the biosynthesis of L-Lactic acid, L-malic acid, fumaric acid, and ethanol, with pyruvate serving as a central intermediary that leads to both the desired organic acids and their by-products. Key enzymes such as pyruvate decarboxylase (PDC), pyruvate dehydrogenase (PDH), alcohol dehydrogenase (ADH), pyruvate kinase (PK), lactate dehydrogenase (LDH), phosphoglycerate kinase (PGK), phosphoglycerate mutase (PGM), glyceraldehyde-3-phosphate dehydrogenase (GPDH) play vital roles in the formation of these metabolites. These enzymes are essential for driving metabolic reactions, which ultimately influence the yield and distribution of the final products. On the other hand, the conversion of xylose to LA hasn't been explored as thoroughly as that of glucose, but studies on the respiration process and xylitol production during xylose utilization by *R. oryzae* indicate that the fungus likely follows an oxidative-reductive pathway for xylose metabolism. This pathway may be similar to the oxidative-reductive processes observed in xylose-fermenting yeasts like *Pichia stipitis, Candida shehatae, and Pachysolen tannophilus* (Maas et al. 2006). More research on xylose metabolism in *Rhizopus* species is necessary to improve the utilization of lignocellulosic biomass.

#### Glycolysis: the central pathway

The production of LA in fungi starts with glycolysis, a fundamental metabolic pathway that converts glucose into pyruvate through a series of enzymatic reactions (Fig. 2) (Kavanagh 2018). Important steps in fungal glycolysis consist of:

- *Phosphorylation of Glucose:* Hexokinase catalyzes the conversion of glucose to glucose-6-phosphate that consumes ATP (1 ATP per glucose).
- *Isomerization and Cleavage:* Phosphoglucose isomerase converts glucose-6-phosphate to fructose-6-phosphate. Then, phosphofructokinase (PFK) catalyzes Fructose-6-phosphate to fructose-1,6-bisphosphate, which is then cleaved into two triose phosphates and DHAP by aldolase.

*Energy generation:* The triose phosphates undergo a series of enzymatic reactions that result in the production of pyruvate, ATP, and NADH. G3P is initially oxidized and phosphorylated to form 1,3-bisphosphoglycerate (1,3-bisPG), with the enzyme GPDH facilitating this reaction. This process also generates NADH from NAD+, which is essential for energy production and maintaining redox balance. The high-energy 1,3-bisPG then undergoes substrate-level phosphorylation, leading to ATP production through the action of PGK. After that, the molecule is converted into phosphoenolpyruvate (PEP). In the final step of glycolysis, catalyzed by PK, PEP is transformed into pyruvate, yielding an additional 2 ATP molecules. Overall, this pathway results in a net gain of 2 ATP per glucose molecule (4 ATP generated, 2 consumed).

#### Lactic acid fermentation: conversion of pyruvate to lactic acid

Significant progress has been made in understanding how *Rhizopus* sp. generates LA. Research has uncovered three LDH enzymes in *R. oryzae* cultures, which consist of one NAD-independent LDH and two NAD-dependent LDH isozymes. One of the NAD + -dependent enzymes plays a key role in converting pyruvate to lactate, although it shows minimal activity in the reverse reaction (Abdel-Rahman et al. 2013). This enzyme is mainly produced during the initial phases of growth and LA production. The second NAD-dependent LDH, aids in converting l-lactate back to pyruvate and is expressed when the culture is grown on non-fermentable substrates such as glycerol, ethanol, and lactate (Zhang et al. 2007a). Under anaerobic conditions or when oxygen is limited, pyruvate is converted into LA by LDH to regenerate NAD + and maintain glycolysis. In this step, LDH catalyzes the reduction of pyruvate to lactic acid. During this reaction, NADH is oxidized back to NAD+, which is crucial for sustaining the glycolytic pathway. This allows glycolysis to continue, even under anaerobic conditions.

*Energy generation:* The reduction of pyruvate to lactic acid regenerates NAD+ from NADH, ensuring that glycolysis can proceed without running out of NAD+. This NAD+ regeneration is essential for maintaining the cell's redox balance and continuing ATP production through glycolysis.

#### Competing pathways: byproduct formation

In the fermentation pathway for LA formation by *R. oryzae*, byproducts can form due to various metabolic pathways that branch off from glycolysis and fermentation processes. These byproducts may vary depending on the conditions of the fermentation (e.g., oxygen availability, nutrient concentration). Under anaerobic conditions, *R. oryzae* predominantly produces LA as the main fermentation product, along with other potential byproducts like ethanol, acetic acid, and formic acid under certain conditions.

- Lactic Acid (from pyruvate via LDH, main product)
- Ethanol (from pyruvate via PDC and ADH, common in some fermentations)
- Acetic acid (from acetyl-CoA, potentially a side product)
- Formic acid (possible byproduct under anaerobic conditions)
- CO_2_ (released during various decarboxylation steps)

Here, ethanol is produced in some *Rhizopus* strains, and acetate, formic acid, and CO_2_ are produced depending on metabolic branching. However, Zhang et al. found that fumaric acid and ethanol are two major by-products of lactic acid fermentation by *Rhizopus* species under aerobic conditions. (Zhang et al. 2007b). Researchers have attempted to minimize the production of these by-products through molecular genetic techniques. Increasing LA production involves limiting ethanol accumulation in *Rhizopus* species by focusing on enzymes such as PDC and ADH. Skory et al. (2004) developed a mutant strain of *R. oryzae* with decreased ADH activity in anaerobic environments, resulting in nearly a tenfold increase in LA production compared to the wild-type strain. Even with reduced ADH activity, this mutant still generated considerable amounts of ethanol under anaerobic conditions. Furthermore, it has been demonstrated that adding Na_2_CO_3_ can boost the production of LA, malic acid, and fumaric acid while suppressing ethanol formation. The highest yield of LA was achieved with 10 mM Na_2_CO_3_, unlike higher concentrations of 20 and 30 mM, indicating that pyruvate carboxylase may compete more effectively for the available pyruvate at this level (Saito et al. 2004).

## Substrates for lactic acid production using fungi

**Fig. 3:**
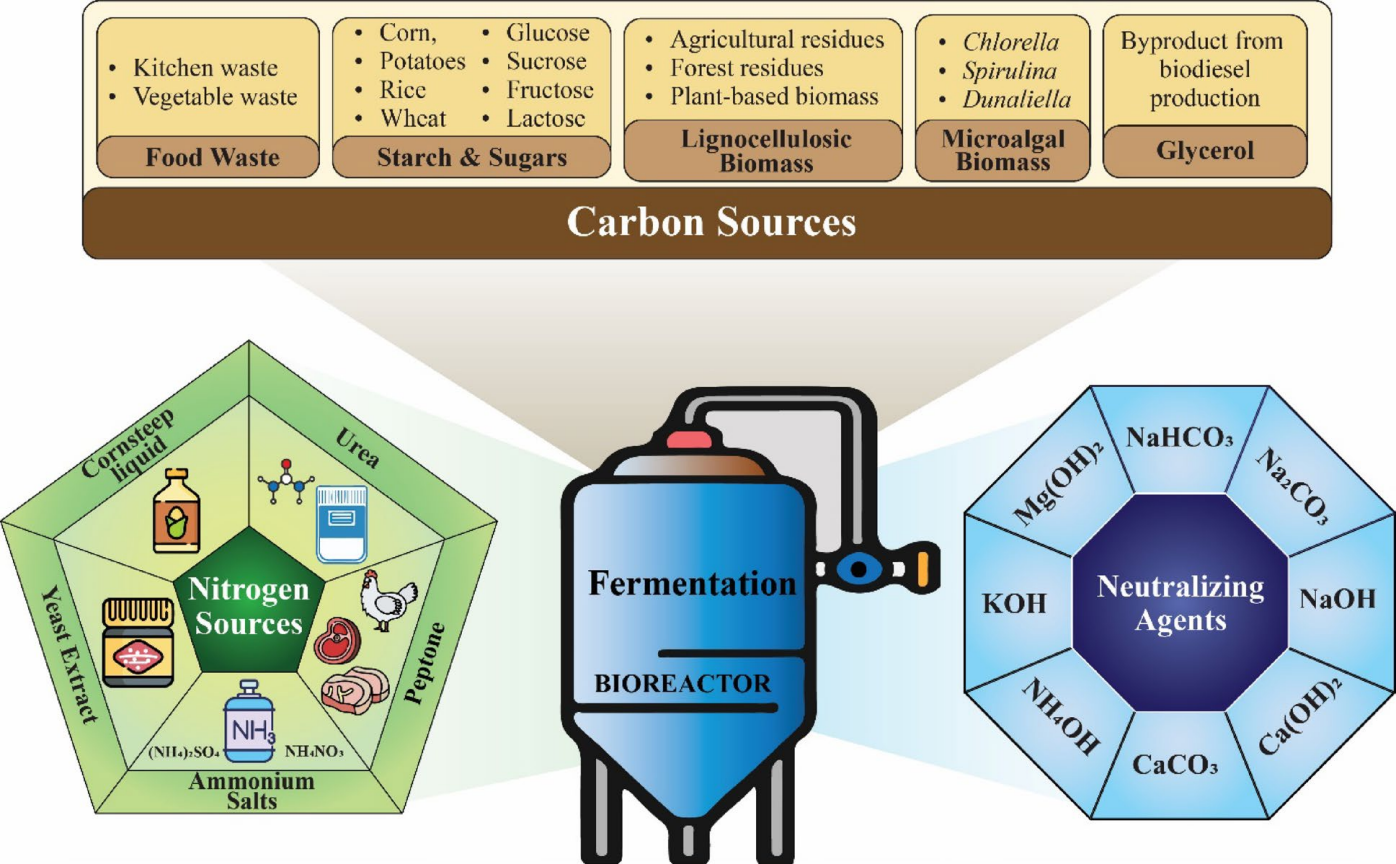
Substrates and additives used in lactic acid production by *Rhizopus oryzae*

For efficient LA production by fungi like *R. oryzae*, selecting the right substrates is vital to maximize yields. Key factors include the type of carbon and nitrogen sources and neutralizing agents, all of which significantly influence fermentation efficiency. Figure 3 illustrates the different substrates used in LA fermentation by *R. oryzae*.

### Carbon sources

The type of carbon source is a critical determinant in the efficiency of LA production by fungi (Dörsam et al. 2017). Several substrates have been explored for LA production, including glucose, lactose, and dairy processing residues like skim milk, and whey. Starch-based materials such as potato, cassava, wheat, rice, and sorghum, as well as molasses and glycerol, have also shown promising results (Meussen et al. 2012). *R. oryzae*, in particular, demonstrates the versatility to metabolize a wide range of carbon sources, including glycerol, ethanol, sugars, fatty acids, and oils, making it highly adaptable for LA production using diverse feedstocks (Dulf et al. 2018; Dhandapani et al. 2021; Sadaf et al. 2021). Table 2 summarizes recent studies on lactic acid production from renewable materials under optimal conditions.

**Table 2:** Recent studies on lactic acid production from renewable materials: concentration, yield, and productivity under optimal conditions across different fermentation methods

Fungal Strain	Renewable Substrate	Fermentation Mode	Optimum Parameters	Lactic Acid (g/L)	Yield (g/g substrate)	Productivity g/(L/h)	References
*R. oryzae* TS-61	Molasses, Chicken feather protein (CFP) hydrolysate	Solid-state fermentation (SSF) [Batch]	Temp. (30 °C), pH (5.5), Inoculum size (5–10%), Incubation time (42 h)	38.5	0.51	–	Taskin et al. (2012)
*R. oryzae* NRRL 395	Solid pineapple waste (SPW)	Solid-state fermentation (SSF) [Batch]	Temp. (32.2 °C), pH (5.6), Moisture content (67.53% w/w), Incubation time (3 days), Inoculum size (1 × 10^7^ spores/g), and C/N ratio (19:1)	41.5	0.104	1.15	Zain et al. (2021)
*R. oryzae*	Solid pineapple waste (SPW)	Solid-state fermentation (SSF)	Temp. (27 °C), pH (6.5), Moisture content (80% w/w), Inoculum size (1 × 10^4^ spores/g), and Incubation time (72 h)	39.5	0.0236	0.0009863	Aziman et al. (2015)
*R. oryzae* CICC41411	Distillers grains hydrolysates (DGHs)	Solid-state fermentation (SSF)	pH (6.0), Inoculation size (2.5% seed culture), CaCO_3_ addition (80 g/L), Incubation time (96 h)	10.88	0.53	0.92	Ren et al. (2013)
*R. oryzae* MTCC5384	Industrial waste paper sludge (PS)	Simultaneous saccharification & fermentation (SSF)	Temp. (40 °C), pH (6.0), Substrate concentration (75 g/L) and Incubation time (144 h)	27	0.8	1	Dhandapani et al. (2021)
*R. oryzae* NRRL-395	Wheat wastewater & glucose	Submerged fermentation (SmF)	Temp (30 °C), pH (6.0), Inoculum size (1.0 × 10^6^ spores/mL), Agitation speed (150 rpm), and Incubation time (8 days)	5.804	0.7	0.75	Göçeri̇ et al. (2021)
*R. oryzae* DMKU 12	α-amylase-treated liquefied Cassava pulp	Submerged fermentation (SmF) [Repeated Batch]	pH (6.0), Inoculum size (0.25 × 0.25 × 0.25 spores/cm^3^), aeration rate (0.75 vvm), and incubation time (72 h)	83.7	0.62	1.16	Trakarnpaiboon et al. (2017)
*R. oryzae* BCRC 33071	Glucose	Solid-state fermentation (SSF) [Batch]	Temp. (30 °C), pH (5.5), and incubation time (96 h)	102.4 ± 1.3	0.85	3.01 ± 0.05	Wang et al. (2014)
*R.oryzae* LA-UN-1	*Zizania latifolia* waste and cane molasses	Batch and Fed-batch fermentation	Temp. (30 °C), pH (5.5), Agitation speed (250 rpm), Inoculum size (10^7^–10^8^ spores/mL), and Incubation time (86 h)	129.47	0.72	1.51	Yin et al. (2023)
*R. arrhizus* DAR 36017	Liquified waste potato starch	Submerged fermentation (SmF)	Temp. (30 °C), pH (6.0), Aeration rate (0.4 vvm), Inoculum size (10^5^spores/mL), and Incubation time (72 or 84 h)	103.8	0.88	2.16	Zhang and Jin (2010)
*R. arrhizus*	Carrot syrup	Submerged fermentation (SmF)	Temp. (30 °C), Agitation speed (150 rpm), pH (6.92), Inoculum size (10^6^ conidia/mL), and Incubation time (72 h)	22.18	0.79	0.31	Salvañal et al. (2021)
*R. oryzae* NRRL395	Cassava pulp hydrolysates	Batch fermentation	Temp. (30 °C), pH (6.0), Inoculum size (10^6^ Spore/mL), and Incubation time (96 h)	75.28	0.5	1.05	Pimtong et al. (2017)
*R. oryzae* 3.819	Sophora flavescens residues	Simultaneous Saccharification and Fermentation (SSF) [Batch]	Temp. (28 °C), pH (6.5), Agitation speed (180 rpm), Moisture content (68.4 ± 3.7%), and Incubation time (24 h),	46.78	–	0.97	Ma et al. (2020)
*R. oryzae*	Yam peel hydrolysate	Batch surface and submerged fermentation (SmF)	Temp. (35 °C), pH (6.5 ± 0.5), Agitation speed (200 rpm), and Incubation time (96 h)	64.02	0.8	–	Ajala et al. (2021)
*R. oryzae*	Glucose	Continuous Batch Fermentation	Temp. (32 °C), pH (5.3), Agitation speed (180 rpm), Incubation time (240 h), Inoculum size (105 spores/mL), and Aeration rate (5 dm^3^/min)	–	–	2.91	Thongchul et al. (2010)
*R. oryzae* GY18	Glucose	Solid-State Fermentation (SSF)	Temp. (35 °C), Incubation time (72 h), Inoculum size (10^6^ spores/mL), and Agitation speed (180 rpm)	115	0.81	1.67	Guo et al. (2010)
	Sucrose		Temp. (35 °C), Incubation time (96 h), Inoculum size (10^6^ spores/mL), and Agitation speed (180 rpm)	80.1	0.89	1.67	
	Xylose		Temp. (35 °C), Incubation time (120 h), Inoculum size (10^6^ spores/mL), and Agitation speed (180 rpm)	54.2	0.9	0.57	
*R. oryzae* NBRC 5378	Xylose	Batch fermentation	Temp. (30 °C), pH (3.5), Agitation speed (300 rpm), and Incubation time (12 h)	14.4	–	0.56	Saito et al. (2012)
*R. oryzae* AS 3.819	Glucose	Semicontinuous fermentation	Temp. (32 °C), pH (5.4–6.0), Agitation speed (200 rpm), Aeration rate (1.0 l/min), Inoculum size (5 × l0^6^ spores/mL), and Incubation time (24 h)	103.7	–	2.16	Wu et al. (2011)
*R. oryzae* NBRC 5384	Glucose	Batch fermentation	Temp. (37 °C), Agitation speed (120 rpm), Inoculum size (2 × l0^5^ spores/mL), and Incubation time (30 min)	145	0.95	1.42	Yamane and Tanaka (2013)
		Fed-batch fermentation	Temp. (37 °C), Agitation speed (120 rpm), Inoculum size (2 × l0^6^ spores/mL), and Incubation time (24 h)	231	0.93	1.83	
*R. oryzae* As 3.819	Glucose	Batch fermentation	Temp. (32 °C), pH (4.7), Agitation speed (180 rpm), Aeration rate (0.5 vvm), Inoculum size (l0^6^ spores/mL), and Incubation time (72 h)	80.2	0.67	4.48	Zhao et al. (2015)
*R. oryzae* NRRL 395	Cassava pulp	Solid State fermentation (SSF)	Temp. (30 °C), pH (6.0), Agitation speed (150 rpm), Inoculum size (10^7^ spores/mL)	463.18	0.83	2.76	Phrueksawan et al. (2012)
*R. oryzae* NRRL395	Cassava pulp hydrolysates	Batch fermentation	Temp. (30 °C), pH (6.0), and Inoculum size (10^6^ spores/mL)	75.28	0.5	1.05	Pimtong et al. (2017)
*R. arrhizus* As 3.3462	Honeycomb matrix	Batch fermentation	Temp. (32 °C), pH (6.0), Agitation speed (200 rpm), Aeration rate (0.5 vvm), Inoculum size (106 spores/mL), and Incubation time (2 d)	68.8	0.93	0.72	Wang et al. (2010)
*R. oryzae* NRRL395	Glucose	Batch fermentation	Temp. (30 °C), pH (6.22), Agitation speed (387 rpm), Aeration rate (0.5 vvm), Inoculum size (10^6^ spores/mL), and Incubation time (7 d)	75.1	0.63	1.54	Coban and Demirci (2016)
*R. oryzae* NRRL396	Pretreated dairy manure	Submerged Fermentation (SmF) [Batch]	Temp. (120 °C), pH (10), C/N ratio (15/1)	1.2	–	–	Sun et al. (2012)
*R. arrhizus* NLX-M-1	Xylo-oligosaccharides manufacturing	Solid State fermentation (SSF)	Temp. (40 °C), pH (> 6.0), Agitation speed (170 rpm), and Incubation time (2–3 d)	34–60.3	0.34–0.60	–	Zhang et al. (2015)
*R. oryzae* 3.819	Animal feeds from Sophora flavescens residues	Simultaneous Saccharification and fermentation (SSF)	Temp. (28 °C), pH (6.5), Agitation speed (180 rpm), and Incubation time (48 h)	46.78	–	0.97	Ma et al. (2020)
*R. microsporus* DMKU 33	Liquefied cassava starch	Batch fermentation	Temp. (40 °C), pH (5.5), Agitation speed (200 rpm), Aeration rate (0.75 vvm), Inoculum size (10^7^ spores/mL), and Incubation time (3 d)	84.3–119	0.84–0.93	1.25	Trakarnpaiboon et al. (2017)
*R. arrhizus* DAR 36017	Waste potato starch	Submerged Fermentation (SmF) [Batch]	Temp. (30 °C), pH (6.0), Agitation speed (200 rpm), Aeration rate (0.4 vvm), Inoculum size (10^7^ spores/mL), and Incubation time (48 h)	103.8	–	2.2	Zhang and Jin (2018)
*Monascus ruber* CBS	Glucose	Fed-batch fermentation	Temp. (35 °C), pH (3.8), Agitation speed (250 rpm), and Incubation time (4 d)	190	0.72	1.15	Weusthuis et al. (2017)
503.7			Temp. (35 °C), pH (2.8), Agitation speed (250 rpm), and Incubation time (4 d)	129	0.58	0.91	
*Aspergillus brasiliensis* BRFM1877	Xylose, Arabinose, Starch and Xylan	Submerged Fermentation (SmF) [Batch]	Temp. (30 °C), pH (3.0), Supplementation with glucose (20 g/L), Agitation speed (140 rpm), Inoculum size (10 spores/mL), and Incubation time (96 h)	32.2	–	0.47	Liaud et al. (2015)
*Aspergillus niger* NCIM 565	Glucose	Submerged Fermentation (SmF) [Batch]	Temp. (37 °C), pH (6.0), and Incubation time (3 d)	7.7	–	0.13	Dave and Punekar (2015)

#### Food waste

It comprises different substances like food production waste and waste by the consumers. Its composition is rich in carbohydrates, particularly sugars, and starches, which makes it a suitable carbon source for LA production. Such are encountered in kitchen waste and municipal solid waste that may be composed of fish, vegetables, meat, cereals, and water (Zhang et al. 2008a, b). Specific examples are cooked rice, bean curd, rice, noodles, meat, vegetable and fruit waste comprising carrot peels, cabbage, potato peels, banana peels, apple skins, orange peels as well as powdered fish and rice, and tea waste (Kwan et al. 2015; Ma et al. 2020).

#### Starch-based materials

Starch-based materials like cassava, potatoes, and various grains (such as wheat, corn, and rice) are commonly used as carbon sources for producing LA because of their carbohydrate content. Cassava starch is especially notable for fermentation by *R. oryzae* NRRL 395 (Thongchul et al. 2010). Additionally, potato pulp and starch byproducts have been effectively utilized, with research showing LA production by both *R. oryzae* and *R. arrhizus* (Zhang and Jin 2008; Akoetey and Morawicki 2018). The use of amylases is crucial for breaking down starch, which aids in the production of optically pure L(+)-LA in mineral media (Wang et al. 2010; Saito et al. 2012). Many studies have utilized simultaneous saccharification and fermentation (SSF) processes for LA production from starch substrates, which enhances the efficiency of converting polysaccharides into LA (Göçeri̇ et al. 2021).

#### Lignocellulosic biomass

Lignocellulosic waste is an excellent feedstock for producing LA because it is renewable, cost-effective, and readily available. This type of waste consists of cellulose, hemicellulose, and lignin, providing a rich source of fermentable sugars that serve as an effective carbon source for fermentation processes (Saito et al. 2012). Agricultural byproducts like straw and wood chips are prime examples of lignocellulosic feedstocks that are both economically and environmentally beneficial. The ability of *Rhizopus* species to efficiently ferment these materials further highlights their potential in sustainable biomanufacturing. Research has identified various lignocellulosic materials suitable for LA fermentation. Zhang and Jin assessed substrates such as rice bran, wheat bran, sugarcane bagasse, and oil cakes, finding that sugarcane bagasse produced the highest LA yield (Zhang and Jin 2008). Likewise, corncobs (Guo et al. 2010), grape stalks, and rice straw (Chen et al. 2018) have been effectively used as a feedstock for LA fermentation. Although *R. oryzae* can utilize xylose, research by Maas et al. indicated that yields were lower compared to those from glucose fermentation, highlighting the need for further optimization (Maas et al. 2006). Improvements in pretreatment and enzymatic hydrolysis have enhanced the accessibility of lignocellulosic sugars (Wang et al. 2014; Göçeri̇ et al. 2021). Zhang et al. used sulfuric acid impregnation and steam explosion on wood before fermenting it with *R. oryzae* NRRL 395 (Zhang et al. 2008a, b). Corncobs, which are rich in cellulose and hemicellulose, were used to produce lactic acid by *R. oryzae* NRRL 395 (Lian et al. 2020) and *Rhizopus sp*. MK-96-1196 (Sadaf et al. 2021) after pretreatment with 0.1 N NaOH solution (Cao et al. 2022).

#### Microalgal biomass

Algal biomass is an attractive substrate for fungal fermentation due to its high carbohydrate content, particularly starches and polysaccharides. Unlike lignocellulosic biomass, algal biomass lacks lignin, simplifying the conversion process and improving efficiency (Nguyen et al. 2012; Ahirwar et al. 2024). Several studies have demonstrated the potential of using algal biomass, such as hydrolyzed sugars from macroalgae like *Laminaria* and *Sargassum*, for LA production (Varman et al. 2013; Lin et al. 2020). Fungal strains like *R. oryzae* have been shown to effectively convert *Chlorella vulgaris* sugars into L-Lactic acid (Agwa et al. 2022). In particular, microalgal hydrolysates, derived from species like *Gracilaria* sp., also serve as excellent carbon sources, leading to substantial LA yields when used in fungal fermentation processes (Kwan et al. 2015).

#### Glycerol

Glycerol, a byproduct of biodiesel production, serves as a cost-effective and renewable carbon source for fungal fermentation and waste valorization strategies. When conditions are optimized, fungi like *R. oryzae* can effectively convert glycerol into LA. Numerous studies have documented the direct production of LA from glycerol by microorganisms, showcasing its potential in sustainable bioprocessing (Vodnar et al. 2013; Papanikolaou et al. 2017; Groff et al. 2024). Crude glycerol has been successfully used in LA production. For instance, *R. oryzae* NRRL 395 grown in media with crude glycerol and supplemented with nutrients such as lucerne green juice (LGJ) produced significant amounts of fungal biomass and L-LA. This illustrates the practicality of utilizing low-cost substrates for generating value-added products (Vodnar et al. 2013). In another investigation, controlled fermentation with different glycerol concentrations and temperatures identified optimal conditions for enhancing LA production. Lower temperatures supported fungal growth, while higher temperatures promoted increased LA synthesis, highlighting the need to balance growth and production phases for better yields (Dulf et al. 2018).

### Nitrogen sources

Nitrogen is an essential nutrient for fungal metabolism and growth, significantly affecting both biomass production and LA yield. It is a key element in the synthesis of amino acids, pyrimidines, purines, some carbohydrates and lipids, co-factors, and other nitrogenous compounds that are vital for cellular functions. To enhance LA fermentation by fungi, various nitrogen sources, both inorganic and organic, are utilized. Commonly used nitrogen sources include inorganic salts (e.g., ammonium sulfate ((NH_4_)_2_SO_4_) and ammonium nitrate (NH_4_NO_3_) and organic compounds like peptone, yeast extract, and corn steep liquor, soybean, peanut, cottonseed, and fish meal. Inorganic nitrogen sources such as ammonium are preferred for fungal microorganisms due to their reduced nitrogen form, which can be readily assimilated into organic compounds (Neu et al. 2016). Among the nitrogen sources available, (NH_4_)_2_SO_4_ is particularly notable for its widespread use, as it effectively supports fungal growth and LA production. Studies on *Rhizopus* species have demonstrated that it can be effectively applied at concentrations between 1.0 and 4.0 g/L in fermentation media (Zhang and Jin 2008; Nguyen et al. 2012; Akoetey and Morawicki 2018). Comparative studies reveal that (NH_4_)_2_SO_4_ outperforms other nitrogen sources, including NH_4_NO_3_, urea (Zhang et al. 2007a), and combinations of urea with yeast extract (Juturu and Wu 2016), in terms of LA production. Organic nitrogen sources like peptone, yeast extract, and corn steep liquor are crucial in the fermentation process. Yeast extract, which is abundant in B vitamins, has been found to boost LA production, but it can be quite expensive, significantly impacting overall production costs in industrial settings (Wang et al. 2010). On the other hand, corn-steep liquor serves as a more affordable option, providing a nutrient-rich mix of soluble proteins, amino acids, and vitamins, making it a suitable organic nitrogen source for LA fermentation (Neu et al. 2016).

Fungi exhibit a distinct advantage in nitrogen utilization, as they can assimilate inorganic nitrogen sources (e.g., ammonium salts) into organic compounds, reducing their reliance on expensive organic nitrogen supplements (Lopes and Ligabue-Braun 2020; Garuba et al. 2022). In contrast, bacterial systems typically require higher amounts of pre-processed organic nitrogen sources, increasing production costs (Kitpreechavanich et al. 2016). While renewable alternatives like red lentil flour or wheat gluten combined with minimal yeast extract have shown promise in bacterial lactic acid fermentation (Altaf et al. 2005, 2007), fungal systems could further lower costs by minimizing or even eliminating the need for organic nitrogen, depending on strain adaptability (Astudillo et al. 2023). Therefore, the choice between fungal and bacterial fermentation should carefully consider nitrogen source flexibility and overall process economics, as fungal-based LA production offers the potential for a more economical and sustainable alternative when paired with affordable or renewable nitrogen sources.

## Different fermentation modes used in lactic acid production

Selecting the optimal fermentation mode is paramount for maximizing yield and economic viability in fungal LA production. This choice can be influenced by several factors, including the type and nature of the substrate, microbial growth characteristics, and the viscosity of the fermentation broth (Ahmad et al. 2020). This section delves into the four dominant fermentation modes (batch, fed-batch, continuous, and cell immobilization), exploring their advantages and limitations for LA production. The comparison among all of these fermentation modes is described in Table 3, while Table 2 summarizes recent studies on LA production using various fermentation methods.

**Table 3:** Comparison among different fermentation modes highlighting their advantages, drawbacks, and lactic acid production performance

Fermentation Mode	Description	Advantages	Disadvantages
Batch	All nutrients are added at the start, and no further feeding occurs during fermentation	Simple operation Low risk of contamination High cell concentration	Substrate and product inhibition Diminishing yield as fermentation progresses
Fed-Batch	Nutrients are added at regular intervals throughout the fermentation process	Less substrate inhibition Increased cell concentration Higher lactic acid yield Better control over nutrient addition	Product inhibition Difficulties in maintaining process conditions Complexity in monitoring and controlling nutrient supply
Repeated Batch	Inoculum from previous fermentation cycles is reused in the next cycle to continue fermentation	Shorter process time Increased cell growth Reduced seed preparation time Higher cell concentration	Decreasing productivity with increasing batch number Problems with cell viability and stability Possible accumulation of unwanted byproducts
Continuous	Fresh medium is continuously added at the same rate while existing broth is removed	Controlled growth High productivity Continuous nutrient replenishment Stable process High product concentration	Incomplete substrate utilization Risk of cell wash out or product accumulation Higher risk of contamination
Separate Hydrolysis & Fermentation	Hydrolysis of the substrate is performed separately before fermentation, allowing optimization of each process independently	Each process is performed at optimal conditions Increased productivity Low enzyme intake	Higher risk of contamination Increased inhibition from byproducts Requires more equipment Careful management required
Simultaneous Saccharification and Fermentation	Required for complex substrates, it is not a standalone fermentation mode and can be combined with any of the fermentation modes (batch, fed-batch, repeated batch, continuous)	Shorter time Reduced reactor volume Reduced inhibition Lower cost	Difficulties in matching optimal conditions for both saccharification and fermentation Lower efficiency

### Batch fermentation

Batch fermentation is the simplest approach. Here, all substrates are added to the fermenter at the outset, and the final product is recovered at the end. Neutralizing agents are often employed during fermentation to maintain optimal pH. This closed system offers a low risk of contamination, facilitating the production of high LA yields. Additionally, batch fermentation achieves complete utilization of the initial substrates, potentially leading to the highest conversion rate among the three modes (Ahmad et al. 2020,Liu and Miao 2020). However, this method suffers from low cell concentration due to limited nutrient availability later in the process. Furthermore, productivity is hampered by substrate or product inhibition as the fermentation progresses (Abdel-Rahman et al. 2013). Different fermentation strategies, including separate hydrolysis and fermentation (SHF), solid-state fermentation (SSF), and simultaneous saccharification and fermentation (SSF), can be applied in batch, fed-batch, and continuous modes to improve lactic acid production. Compared to SHF, SSF offers several advantages: a single reactor setup, faster processing times, reduced inhibitory effects on hydrolysis, and lower enzyme requirements (Abdel-Rahman et al. 2015).

### Fed-batch fermentation

Fed-batch fermentation addresses some of the limitations of batch fermentation by introducing substrates sequentially without removing the fermentation broth (Ding and Tan 2006). In fed-batch fermentation, the amount of limiting nutrients such as carbon and nitrogen added sequentially at regular intervals determines the reaction rates. In addition, adding limiting nutrients enhances microbial growth, resulting in higher yield. Compared to batch fermentation, this method has advantages. Liu et al. discovered that in fed-batch culture, approximately 140 g/L of L-LA was produced with an 83% product yield when the glucose concentration was kept at 30 g/L (Liu et al. 2006). In contrast, batch fermentation produced a reduced yield and only 121 g/L of L-LA when the initial glucose content was 200 g/L. Using fed-batch fermentation, also reported reaching a maximum LA production and productivity to be 162 g/l and 6.23 g/l/h (Fu et al. 2018).

### Repeated fermentation

Repeated fermentation, whether using batch or fed-batch modes, involves inoculating a portion or all of the cells from a previous fermentation cycle into the next one (Zhao et al. 2015). Various methods are used for cell recycling in bacteria and fungi. For bacteria, techniques such as centrifugation, hollow fiber modules, or using part of the culture. For fungi, methods like filtration or mycelial pellet precipitation are typically employed. Compared to batch or fed-batch fermentation (Table 3), repeated fermentation offers several benefits, including higher yield, less time and labor required to clean and sterilize the fermenter, no need to prepare seeds, higher cell concentrations, higher LA productivity, and shorter fermentation times because of large initial inoculation volumes (Abdel-Rahman et al. 2013). Many studies have investigated the use of *R. oryzae* in repeated fermentation for the generation of L-LA. Yin et al. used tiny mycelial pellets of *R. oryzae* NRRL395 to produce 2.02 g/L/h of LA from maize starch over nine cycles in 14 days in an air-lift bioreactor. This was 1.9 times greater than in batch fermentation (Yin et al. 2023). Jin et al. examined filamentous and pellet forms of *R. arrhizus* 36,017 and *R. oryzae* 2062, obtaining productivities of 5.06 and 4.39 g/L/h, respectively (Jin et al. 2005). Using the floc-form of *R. oryzae*, Yu et al. (2007) performed repeated batch fermentation over six cycles, achieving a maximum productivity of 4.03 g/L/h. Liu et al. used pelletized *R. oryzae* NRRL 395 to form a method for coproducing LA and chitin, which produced 66 g/L LA concentration (Liu et al. 2005). Further research on fungal growth and metabolic processes during repeated fermentation is needed to understand its impact on microorganism growth and product formation (Wu et al. 2017).

### Continuous fermentation

Continuous fermentation maintains constant substrate and product concentrations by continually adding fresh medium to the fermenter and simultaneously removing the existing broth, which contains the used medium and cells, at the same rate. This process ensures the replenishment of consumed nutrients and the removal of toxic metabolites from the culture (Ahmad et al. 2020). While using semi-continuous fermentation with *R. oryzae* AS 3.819, Wu et al. obtained LA concentration of 103.7 g/L with a productivity of 2.16 g/(L/h) (Wu et al. 2017). According to a different study by Pimtong et al. employing a static bed fermentor with complete immobilization of *R. oryzae* on a fibrous matrix achieved a LA productivity of 1.05 g/(L/h), with a final titer of 75.28 g/L. The key advantage of this setup is improved mass transfer and operational ease due to cell-free broth, while its strength also lies in high tolerance to salt impurities and suitability for continuous fermentation without cell washout (Pimtong et al. 2017).

### Solid-state fermentation (SSF) and simultaneous saccharification and fermentation (SSF)

Solid-state fermentation (SSF) involves the growth of fungi on moist solid substrates without free-flowing water. This method is particularly suitable for filamentous fungi such as *Rhizopus* species. SSF offers several advantages, including higher product concentration, reduced water usage, and lower risk of contamination compared to submerged fermentation (Taskin et al. 2012; Aziman et al. 2015; Zain et al. 2021). Substrates used in SSF are typically agro-industrial residues, such as wheat bran, rice straw, or corn stover, which also act as support for fungal growth. SSF has been widely employed to produce LA, enzymes, and other value-added products (Abdel-Rahman et al. 2013). The kinetics of SSF with *Rhizopus* species have been extensively studied. For example, Saito et al. found that SSF yielded 6 g/L of lactic acid from powdered wheat straw, outperforming SHF, which produced only 2 g/L (Saito et al. 2012). Further studies have demonstrated the feasibility of using low-cost feedstocks for LA production. *R. oryzae* CICC41411 efficiently produced lactic acid from distillers grains hydrolysates under optimized SSF conditions (Ren et al. 2013). Groff et al. optimized SSF of grape stalks using *R. oryzae* NCIM 1299 with a variable temperature profile, achieving a 53% increase in final LA concentration, highlighting the potential of SSF to improve productivity (Groff et al. 2022).

Simultaneous saccharification and fermentation (SSF) combines enzymatic hydrolysis of polysaccharides and microbial fermentation in a single step. By integrating these processes, SSF reduces sugar accumulation, lowers enzyme requirements, and improves productivity. Fungal enzymes, produced either by the fermenting organism or added externally, facilitate the conversion of complex carbohydrates into fermentable sugars that are immediately consumed by the microorganism to produce LA. Several studies have demonstrated the effectiveness of SSF in fungal LA production. Ma et al. reported that SSF with *R. oryzae* 3.819 using Sophora flavescens residues produced 46.78 g/L of LA (Ma et al. 2020). *R. oryzae* MTCC5384 produced 27 g/L LA from industrial waste paper sludge at 40 °C, pH 6.0, substrate concentration 75 g/L, and 144 h incubation (Dhandapani et al. 2021), while *R. arrhizus* UMIP 4.77 yielded 10 g/L LA from wheat straw (Vially et al. 2010). Similarly, Yin et al. explored the production of LA from industrial wastepaper sludge using *R. oryzae* MTCC5384 through SSF (Yin et al. 2023). Both SSF and simultaneous saccharification and fermentation represent key strategies in fungal fermentation due to their operational efficiency and suitability for lignocellulosic substrates.

### Cell immobilization

From an industrial standpoint, cell immobilization is a highly effective approach to boost fermenter cell density and improve LA production. This technique offers additional advantages, including enhanced cell stability, reduced dependence on nitrogen sources, increased reusability of cells, and the potential to integrate fermentation with separation processes, thereby minimizing downstream processing. Moreover, the high concentration of immobilized cells significantly lowers the risk of contamination (Zhang et al. 2007b; Gao et al. 2009; Chotisubha-Anandha et al. 2011). However, the possibility of mass-transfer restrictions is a major drawback of immobilized cell systems (Kosseva et al. 2009). Various carriers, fermentation techniques, and fermenter integrations have been studied for the immobilization of *R. oryzae*. Matrices such as calcium alginate beads, cotton cloth, loofa sponge, and polyvinyl alcohol (PVA) cryogel polymers have been found effective for enhancing LA production, though each has specific limitations (Ganguly et al. 2007; Zhang et al. 2008a, b). Bioreactor integration can enhance immobilization efficacy. A variety of bioreactor systems, such as air-lift, drum contactor, reciprocating jet, tower, and hollow fiber bioreactors, have been used to facilitate submerged filamentous fungal fermentations using immobilized *Rhizopus* cells (Gomes et al. 2023; Cerrone and O’Connor 2025). Despite this, many studies report LA yields ranging from 0.65 to 0.78 g/g, with concentrations of 40–73 g/L (Wang et al. 2010; Pimtong et al. 2017). In some cases, yields exceeding 85% have been achieved. However, challenges such as complex operational procedures, support material degradation, and bioreactor damage due to high agitation rates limit the broader application of fungal immobilization (Chotisubha-Anandha et al. 2011). Although many laboratory-scale studies have demonstrated.

high yields and productivity of LA, pilot-scale investigations are necessary to assess the technical and economic feasibility of these processes.

## Optimization of process parameters for *Rhizopus oryzae*

The production efficiency of LA is affected by several key factors. These include the selection of fungal strains, the availability of nutrients (such as carbon and nitrogen sources and their C/N ratios), temperature, pH, incubation time, oxygen levels, and the specific culture conditions used. Table 2 presents the optimal parameters from several recent studies on LA production.

### Fungal strain

The selection of the appropriate fungal strain is crucial for maximizing productivity. Research consistently highlights *Rhizopus oryzae* as a highly effective species for LA fermentation (Thongchul et al. 2010; Göçeri̇ et al. 2021; Zain et al. 2021; Rodríguez-Torres et al. 2022). Its ability to adapt to various conditions and utilize commercial carbon sources makes it an attractive option for sustainable and cost-efficient biochemical production. Specific strains of *R. oryzae* have demonstrated notable advantages in LA synthesis. For example, *R. oryzae* NRRL 395 exhibits remarkable efficiency when fermenting glucose or starchy materials (Pimtong et al. 2017; Zain et al. 2021). Another strain, *R. oryzae* ATCC 9363, is recognized for its versatility in processing diverse substrates, such as agricultural residues, positioning it as a valuable resource for industrial-scale applications (Taherzadeh 2003; Maas et al. 2006)).

### pH

Lactic acid production lowers the pH of the growth media, which can become inhibitory for continued cell growth and fermentation (Uyar et al. 2010). *R. oryzae* is able to withstand higher concentrations of lactic acid at low pH, which is an advantage compared to bacterial systems (Matsumoto 2018). It can grow on a wide pH range of 4.5–7.5, and it has been demonstrated that mycelium formation is increased when the culture has an acidic pH, with the optimal being 3.4–4.5 (Ibarruri and Hernández 2018). One of the most critical parameters to control is the pH and for that purpose, NaOH or CaCO_3_ buffering agents are used frequently (Bai et al. 2003; Ren et al. 2013; Zain et al. 2021). Studies by Ren et al. and Aziman et al., suggest that higher pH values favor increased lactic acid production due to changes in fungal morphology and the breakdown of the reaction equilibrium (Ren et al. 2013; Aziman et al. 2015). The optimal pH range for *R. oryzae* in LA production is 5.5–6.0, as it supports efficient carbohydrate conversion and the activity of key enzymes like lactate dehydrogenase and glucoamylase, resulting in high LA production (Wang et al. 2014). Within this pH range, *R. oryzae* has been reported to produce 83–84 g/L of LA, with a yield of 0.84 g/g total sugar and a productivity of 1.15–1.17 g/L/h (Huang et al. 2005).

### Temperature

Temperature is one of the critical parameters in LA fermentation, significantly affecting fungal metabolism, enzyme activity, and overall LA yields (Maslova et al. 2019; Dhandapani et al. 2021). The ideal temperature range for *R. oryzae* fermentation is between 30 °C and 37 °C, where both enzymatic reactions and fungal growth are at their peak. Deviating from this range can impede metabolic processes, leading to lower LA production (Trakarnpaiboon et al. 2017; Zain et al. 2021). Research has underscored the importance of temperature in optimizing saccharification and fermentation. For example, Ma et al. explored temperatures from 22.5 °C to 40 °C and found that 32.5 °C was the most effective for maximizing LA production from *Sophora flavescens* residues (Ma et al. 2020). Liu et al. determined that 27 °C is the optimal temperature for producing L(+)-LA from cull potatoes using *R. oryzae* NRRL 395 (Liu et al. 2005). In a similar vein, Dhandapani et al. noted an upward trend in LA production with increasing temperature, reaching a peak yield of 21.3 g/L at 40 °C. However, temperatures above 40 °C resulted in a significant drop in LA production, with a 15.4% decrease at 44 °C, which was linked to the thermal inactivation of cellulase and other enzyme active sites. Lower temperatures favor saccharification, while higher temperatures enhance fermentation (Dhandapani et al. 2021). The temperature of 40 °C serves as a crucial transition point, balancing these two processes. Beyond this threshold, enzymatic inefficiency and stress on the fungi can hinder overall productivity.

### Nutrient availability and C/N ratio

The availability and type of nutrients, especially nitrogen, have a significant effect on microbial growth, the production of extracellular enzymes, and the synthesis of LA. To optimize LA production in *R. oryzae* cultures, a limited nitrogen supply is generally recommended (Yu et al. 2007; Thongchul et al. 2010). Inorganic nitrogen sources, such as (NH_4_)_2_SO_4_, are more effective for LA production compared to alternatives like ammonium nitrate, urea, or yeast extract (Jin et al. 2005; Ren et al. 2013). Research indicates that the optimal concentrations of (NH_4_)_2_SO_4_ for *Rhizopus* species fall between 1.0 and 4.0 g/L (Marták et al. 2003; Zain et al. 2021; Zaveri et al. 2022). Likewise, NH_4_Cl, with a nitrogen concentration of 3.5 g/L, is an efficient nitrogen source (Ren et al. 2013). The C/N ratio is also crucial in LA production, affecting substrate utilization and overall yields. The optimum range of C/N ratio was found 10:1 to 20:1; the best LA productivity has been observed at a C/N ratio of 19:1 (mol/mol), with significantly lower productivity at higher ratios, such as 37:1. Although yeast extract is very effective for LA production, its high cost limits its use in large-scale applications. Other alternatives, including hydrolysates from fish waste (Gao et al. 2006), chicken feather protein hydrolysate (CFPH) (Taskin et al. 2012), *Sophora flavescens* residues (Ma et al. 2020), silkworm larvae (Timbuntam et al. 2006) and grape stalk (Groff et al. 2022) have been investigated as cost-effective nitrogen sources. Among these, CFPH is particularly notable for its affordability and capacity to maintain high LA productivity.

### Incubation time

The incubation time affects the efficiency of LA production during fungal fermentation. *R. oryzae* TS-61 showed the best LA production after 72 h of fermentation when using molasses and CFPH as substrates (Taskin et al. 2012). Likewise, research by Aziman et al. and Zain et al.indicated that peak LA yields were achieved at 72 h when fermenting solid pineapple waste (Aziman et al. 2015; Zain et al. 2021). On the other hand, Ren et al. discovered that the highest LA production occurred after 96 h of incubation, followed by a decrease in yields (Ren et al. 2013). Prolonging the incubation time beyond the optimal duration can reduce LA production. Bulut et al. found that the highest LA concentration was reached at 105 h during a 245-h monitoring period, with a gradual decline in LA levels observed afterward (Bulut et al. 2009). These reductions may be due to substrate depletion or metabolic changes in the fungal cells.

### Inoculum type and size

The type of inoculum used for *R. oryzae* impacts growth rate, substrate utilization, and product yield. Common inoculum types include spores (conidial inoculum), hyphal fragments (mycelial inoculum), and combinations of both. Spores are stable and have high germination rates, ideal for submerged fermentations, while hyphal fragments promote faster substrate colonization and reduced lag time, useful in solid-state fermentations. The choice of inoculum affects factors like inoculum density, media, temperature, and aeration. Studies have shown that a 5–10% spore concentration of *R. oryzae* enhances enzyme production (Trakarnpaiboon et al. 2017; Salvañal et al. 2021), while mycelial inoculum optimized solid-state fermentation processes for faster growth and higher yields of LA (Wang et al. 2014; Zain et al. 2021). Meanwhile, optimizing inoculum size is key; too small inoculum can lead to a longer lag phase because microbial activity and colonization are slower, while too large can deplete substrates, accumulate by-products, and hinder oxygen transfer (Ren et al. 2013; Dhandapani et al. 2021). Research has shown that an optimal inoculum size of 5–10% (v/v) for *R. oryzae* is ideal for maximizing LA production with variations based on strain and substrate type. When the inoculum size exceeds 10%, it may lead to more biomass being produced, but this can come at the cost of LA yield, ultimately decreasing fermentation efficiency (Taskin et al. 2012; Zain et al. 2021). For instance, Bulut et al. found that a 7.5% inoculum size was optimal for LA production with glucose-based substrates, while Taskin et al. reported similar outcomes using CFPH (Taskin et al. 2012).

### Oxygen level

*R. oryzae* is known to survive in low-oxygen environments, but it performs best in anaerobic or microaerobic conditions, which are ideal for boosting LA production. However, when oxygen levels increase, the fungus may shift its metabolism to other pathways, producing unwanted by-products instead of LA. Research has demonstrated that no oxygen promotes more effective LA production by *R. oryzae* (Londoño-Hernández et al. 2017; Rodríguez-Torres et al. 2022).

### Neutralizing agents

Neutralizing agents play a crucial role in LA fermentation by maintaining a stable pH in the broth. Some commonly used neutralizing agents include calcium carbonate (CaCO_3_), calcium hydroxide (Ca(OH)_2_), sodium carbonate (Na_2_CO_3_), sodium bicarbonate (NaHCO_3_), sodium hydroxide (NaOH), ammonia (NH_3_), ammonium hydroxide (NH_4_OH), and potassium hydroxide (KOH) (Yen et al. 2010; Zhao et al. 2015). Among them, Ca(OH)_2_ and CaCO_3_ stand out as particularly beneficial because they convert LA into calcium lactate, which helps reduce the toxic effects of LA on the growth and structure of microbial cells (Pleissner et al. 2013; López-Gómez et al. 2020). However, there are some limitations to their use. These calcium-based neutralizers tend to act slowly and have a relatively mild ability to effectively regulate pH (Yen et al. 2010). Additionally, extracting LA from calcium lactate necessitates treatment with sulfuric acid (H_2_SO_4_), resulting in the production of calcium sulfate (gypsum) as a byproduct. This gypsum has limited commercial value, and its disposal can create significant environmental issues (Pal et al. 2009).

Ammonia and its hydroxide are commonly utilized as neutralizing agents, coming in second only to calcium compounds in the context of LA fermentation. The advantages of using ammonia are significant: it effectively manages pH levels, provides an additional nitrogen source that promotes cell growth, and generates (NH_4_)_2_SO_4_ as a byproduct after acid hydrolysis, which is valuable in the fertilizer market (Tian et al. 2014; Wang et al. 2014; Zhao et al. 2015). However, there are important drawbacks to consider. Compounds based on ammonia can be harmful to microbial growth and may induce osmotic stress in the later stages of fermentation due to the buildup of ammonium lactate, which can ultimately lead to a decrease in LA titer (Tian et al. 2014; Wang et al. 2014; Zhao et al. 2015). To address these issues, Liu et al. improved the fermentation process by continuously removing lactic acid through adsorption with poly-4-vinylpyridine (PVP) resin, which enabled a more effective fermentation process (Liu et al. 2006).

## Recent advancements in lactic acid fermentation

### High cell density fermentation

High cell density (HCD) cultivation, which achieves cell concentrations about ten times higher than in traditional batch fermentation, significantly boosts LA productivity, making it ideal for large-scale, cost-effective production. This method reduces contamination risks but poses challenges in design and operation due to its complexity (Chang et al. 2011; Abdel-Rahman et al. 2013). Recent improvements in HCD fermentation, particularly with *R. oryzae*, have enhanced LA yields while addressing economic and environmental issues. Optimizing parameters like pH, temperature, and oxygen supply has accelerated cell growth, increasing LA production. Groff et al. (2024) achieved a 53% increase in LA concentration by *R. oryzae* NCIM 1299, when fermenting grape stalks at 40 °C, with pellet formation enhancing productivity. Pellet density, inoculum size, and nutrient supply were crucial for optimizing yields, with an optimal pellet density of 50–60 kg/m3 achieving up to 57.3 g/L LA (Groff et al. 2024). Fed-batch fermentation, a common method for HCD, has outperformed batch fermentation, achieving LA concentrations of 162 g/L compared to 101 g/L, with reduced processing time and improved efficiency (Fu et al. 2018). Cell immobilization techniques are the most common method for HCD for enhancing cell density and fermentation efficiency. Bioreactor integration can enhance immobilization efficacy. A variety of bioreactor systems, including stirred tank bioreactors (Chotisubha-Anandha et al. 2011), static bed reactors (Pimtong et al. 2017), bubble column bioreactors (Wang et al. 2010), and three-phase fluidized bed bioreactor (Lin et al. 2007) have been used to facilitate submerged filamentous fungal fermentations using immobilized *Rhizopus* cells. These fermenters are designed to optimize aeration, maintain microaerobic conditions (around 0.5–5% oxygen), improve mass transfer, minimize shear stress, and support more consistent cell growth at elevated densities.

Cell recycling systems also help maintain HCD in continuous cultures, allowing for efficient product extraction (Abdel-Rahman et al. 2013). Membrane cell recycling systems, employing ultrafiltration or microfiltration membranes in a semi-closed loop, improve broth homogeneity, enable complete cell recycling, and facilitate simultaneous production and separation of fermentation products. These systems significantly increase cell concentration and LA productivity compared to conventional methods. However, the application of such systems to filamentous fungi like *Rhizopus oryzae* presents unique challenges. The filamentous nature of *Rhizopus* leads to the formation of dense mycelial pellets, which can cause clogging in filtration systems and complicate biomass separation. To address these issues, researchers have explored various strategies (Liu et al. 2006; Lin et al. 2020). For example, Spiricheva et al. investigated the use of immobilized *R. oryzae* cells within polyvinyl alcohol (PVA) cryogels, which not only facilitated biomass retention but also enhanced resistance to high concentrations of accumulated LA, leading to higher yields in iterative fermentation cycles (Spiricheva et al. 2007). Similarly, Efremenko et al. developed immobilized *R. oryzae* cells entrapped in PVA-cryogel beads, achieving increased LA production due to improved resistance to product inhibition (Efremenko et al. 2006). Further advancements include the development of bioreactor designs that mitigate fouling and enhance system stability. Xu et al. employed an electromagnetic flow meter and a pneumatic diaphragm pump, achieving a lactic acid productivity of 31.5 g/L/h with stable operation for over 155 h, outperforming conventional pumps (Xu et al. 2006). Additionally, Ramchandran et al. employed a media-backwash method, boosting LA production by more than twofold by enhancing the performance of submerged hollow fiber membranes (Ramchandran et al. 2012). Ongoing research continues to improve HCD cultivation, advancing the efficiency and sustainability of LA fermentation. These studies underscore the feasibility of adapting cell recycling systems for *Rhizopus*-based LA fermentation. By optimizing fungal morphology, employing suitable immobilization techniques, and implementing advanced bioreactor designs, the challenges associated with cell recycling in fungal systems can be effectively addressed, leading to enhanced productivity and sustainability in industrial-scale operations.

### Advances in downstream processing: recovery and purification of lactic acid from Rhizopus fermentation

Downstream processing of lactic acid (LA) from *Rhizopus*-based fermentations faces unique challenges due to filamentous growth, high mycelial biomass, and viscous broths. The fermentation broth typically contains residual sugars, nutrients, organic acids, pigments, and fungal biomass, an efficient separation and purification are critical for obtaining high- purity LA suitable for industrial and food applications (Ghaffar et al. 2014; Ahmad et al. 2020).

Traditional methods include precipitation, filtration, acidification, carbon adsorption, evaporation, and crystallization (Pal et al. 2009), yet these steps can be influenced by the presence of fungal biomass. Industrial-scale data on fungal LA recovery remain limited (Abdel-Rahman et al. 2013), but several separation and purification techniques have been adapted for fungal systems. Precipitation (Nakano et al. 2012), emulsion liquid membranes (Kumar et al. 2019a, 2019b), solvent extraction (Alkaya et al. 2009; Krzyżaniak et al. 2013), and membrane separation have been explored to overcome challenges posed by complex fungal broths. Membrane-based approaches, including ultrafiltration, reverse osmosis, and electrodialysis, have shown high efficiency in LA recovery while reducing contamination from fungal biomass (González et al. 2008; Kosseva et al. 2009; Din et al. 2021). A hybrid-membrane process achieved 99.5% LA purity, though high membrane costs limit widespread adoption (Lee et al. 2017).

**Fig. 4:**
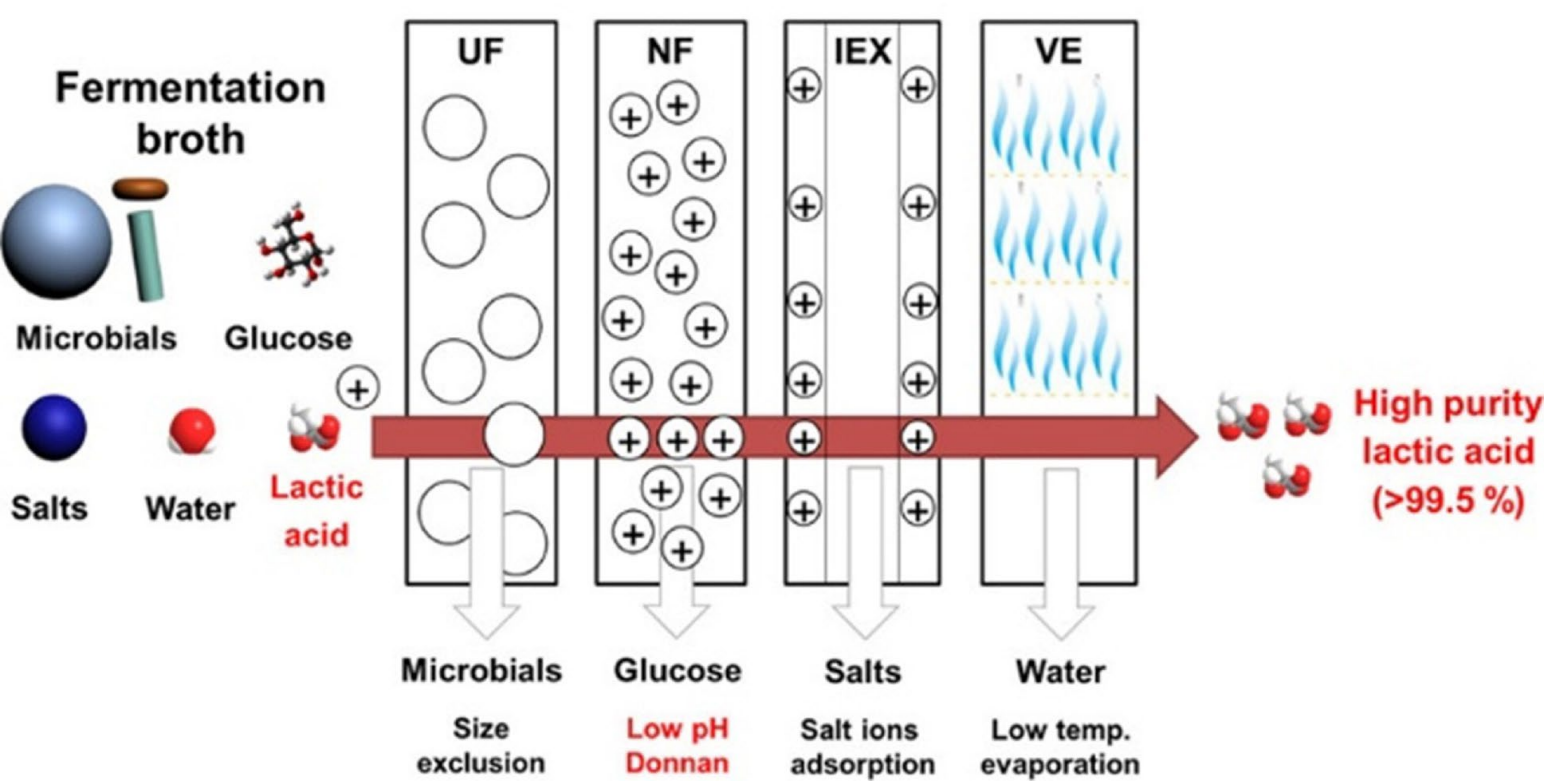
Lactic acid purification techniques: Electrodialysis; (with copyright permission from 2017 American Chemical Society); (Chandukishore et al. 2024)

Further innovations include multi-stage membrane-integrated reactors and multi-pass distillation, achieving LA yields above 96.5% and a purity of 95% (Yu et al. 2007; Pleissner et al. 2013). Combining multiple downstream operations, including nano-filtration, softening, electrodialysis, and ion exchange chromatography, has produced L(+)-LA at 937 g/L with 99.7% purity (Pleissner et al. 2013)(Table 4). Ion exchange has been employed to recover LA with optical purity exceeding 99% (González et al. 2008; Din et al. 2021). Ion-exchange chromatography and two-step extraction methods such as salting-out and reactive extraction have also been successfully applied in fungal LA production (González et al. 2008; Din et al. 2021; Lan et al. 2019). However, challenges such as membrane fouling and high costs limit the widespread adoption of membrane separation and electrodialysis at industrial scales (Komesu et al. 2017). Alternative techniques, including reactive and molecular distillation (Ahmad et al. 2020), as well as the direct fermentation of organic lactates like ammonium lactates, have been explored to produce optically pure LA (Ojo and De Smidt 2023). Among emerging approaches, electrodialysis offers efficient removal of nonionic impurities (Fig. 4) and allows NaOH recycling, which can integrate with fungal fermentation by avoiding calcium salt formation (Juturu and Wu 2016; Wang et al. 2014). Bipolar membrane electrodialysis further improves sustainability by enabling simultaneous separation, purification, and salt-to-acid conversion without generating waste effluents (Vaidya et al. 2005; Kamm 2015).

Specifically, in *Rhizopus oryzae* BCRC 33071 fermentation, ammonia-based neutralization using ammonium bicarbonate and urea achieved a productivity of 3.01 g/L/h and a yield of 85.3%, highlighting its potential for integration with electrodialysis (Wang et al. 2014). Despite these advancements, challenges such as membrane fouling, high operational costs, and ensuring compatibility with fungal fermentation systems remain. Continued research is essential to optimize downstream processes tailored to *Rhizopus*-based LA production, improving both sustainability and industrial feasibility .

**Table 4:** Comparative analysis for more recent downstream studies for lactic acid recovery and purification techniques (Ahmad et al. 2020)

Ranking	Techniques	Advantages	Disadvantages	Recovery (%)	Purity (%)
1st	Electrodialysis	Low operating cost, low energy consumption, high scalability, eco-friendly processes, minimum product inhibitory effect, high degree of separation	Membrane fouling, periodic cleaning, initial investment for setup	85.6	98.9
				81	–
2nd	Membrane methods	Low maintenance cost and high recovery rates, high selectivity, minimal back mixing, ease of scaling up, no direct contact between microbes and toxic solvents, seamless integration with fermenters to reduce costs	High capital cost, challenges in process optimization, membrane fouling issues, and complications arising from polarization	76	99.5
				51.5	99
3rd	Reactive Distillation	Produces high-purity lactic acid; reduced capital and operating costs, single unit process for reaction and separation suitable for integration with other processes	Complicated process, high energy demand leads to elevated operational costs, energy-intensive resulting in high carbon emissions and less feasible for large-scale or sustainable production	92	95.6
				–	99.7
				74.09	95.6
4th	Reactive Extraction	Eco-friendly solvents reduce environmental concerns, solvent recovery reduces recurring costs, high selectivity, ease of operation, and reusability of solvents	Setup is expensive, solvent toxicity concerns, solvent losses, and cost of solvent regeneration	48.81	–
				80	–
5th	Adsorption (Ion-Exchange Resins)	High recovery efficiency, low chemical costs, selective separation, minimal chemical waste, simple regeneration process, and low recurring operational costs	Resin fouling, limited lifetime of resins, poor capacity, resin disposal may generate non-biodegradable waste time-consuming, a higher cost for large-scale use	80	99
				51.5	99
				80	92
6th	Precipitation	Low-cost chemicals, simple to operate, facilitate process optimization, and dependable	Poor product purity, time-consuming, generates significant chemical waste (H_2_SO_4_), less sustainable compared to modern techniques	89.7	71.49
				92	–
				62	–
7th	Emulsion Liquid Membrane	High mass transfer efficiency, reduced energy and chemical usage, more cost-effective than solvent extraction, requires lower capital investment, potential for achieving high recovery rates	Complex operational setup, limited scalability, dependency on petroleum-derived organic solvents	–	97
				–	90
				–	95

## Challenges associated with *Rhizopus*-based lactic acid production

Lactic acid production through fungal fermentation faces several limitations and challenges that affect the efficiency, cost, and scalability of the process. Overcoming these challenges is crucial to optimizing production and establishing fungal fermentation as a viable industrial method. Table 5 outlines the numerous challenges associated with scaling up LA fermentation using fungi.

**Table 5:** Challenges and strategies for scaling up lactic acid fermentation using *fungi*

Challenge	Details/impact on scaling	Strategies
Substrate inhibition	High sugar causes osmotic stress and reduces fungal growth; cellobiose accumulation inhibits cellulase activity, limiting glucose availability	Use fed-batch/continuous fermentation to lower sugar levels, SSF for continuous hydrolysis, ß-glucosidase
Tolerance to inhibitors	By-products like acetic acid and formic acid disrupt fungal membranes, reducing growth	Apply detoxification (e.g., filtration, adsorption with activated charcoal, or biochar), optimize pretreatment, genetically engineer resistant strains
End-product inhibition	LA accumulation lowers pH and damages membranes	Remove LA in situ, use acid-tolerant strains, buffer media for pH stability
Nutrient optimization	Variable feedstock composition and imbalanced C/N ratios hinder fungal growth	Analyze feedstock, optimize nutrients and pH, use controlled agitation for uniform conditions
Morphological variability	Irregular fungal morphology reduces nutrient and oxygen transfer	Control morphology with agitation, modifying agents, optimized bioreactor designs
Downstream processing	High purification costs and by-products complicate recovery	Use in-situ product removal (e.g., electrodialysis, ion-exchange resins, or membrane filtration), greener extraction methods
Scaling-up challenges	Variable morphology (e.g., pellets, filaments) and long fermentation times hinder scalability	Optimize reactor design (e.g., stirred-tank or airlift bioreactors), use morphology modifiers (CaCO_3_), adopt immobilized fungi or biofilm systems
Contamination risk	A high risk of bacterial or yeast contamination in non-sterile environments disrupts the process	Engineer resistant strains, maintain aseptic conditions or use semi-continuous systems
Oxygen transfer	Limited oxygen transfer lowers yield; aeration increases costs	Enhance bioreactor aeration (e.g., spargers, baffles), co-culture microorganisms for oxygen management
Spore consistency	Variable spore quality affects scalability and productivity	Standardize spore protocols (cryopreservation), use pre-adapted spores, and automate inoculum preparation
Slow growth/yield	Slow fungal growth extends fermentation times and lowers LA yields	Optimize parameters, engineer high-yield strains, use co-cultures or immobilized cells

The selection of substrates plays a pivotal role in determining the cost associated with LA production. Numerous low-cost substrates have been tested and proposed for LA fermentation. While some studies have utilized glucose as a substrate, others have found that starches (such as corn starch and potato starch) (Akoetey and Morawicki 2018; Göçeri̇ et al. 2021) and lignocellulosic materials (like agricultural residues and woody biomass) (Chen et al. 2018; Groff et al. 2022) are more cost-effective alternatives. Although agricultural waste and lignocellulosic materials are economically favorable, they can sometimes lead to incomplete fermentation or the formation of undesirable by-products. For instance, lignocellulosic materials can generate phenolic compounds, while carbohydrate-based substrates can produce furans. Both phenolics and furans inhibit lactate dehydrogenase activity and are toxic to *Rhizopus oryzae*, ultimately reducing the purity and concentration of the final lactic acid product (Zhang et al. 2016). Achieving high yield and purity in lactic acid production typically requires careful optimization of fermentation conditions, including pH, temperature, nutrient supply, and oxygen levels. Fungal fermentation generally occurs at acidic pH levels; however, maintaining a stable pH throughout the fermentation process is challenging due to the continuous production of lactic acid, which lowers the pH and can inhibit fungal growth. To mitigate this issue, neutralizing agents like calcium carbonate (CaCO_3_) are often added, but this leads to the formation of lactate salts, which must be reacidified to recover pure LA, adding extra steps and costs to the process. Alternatively, ammonia water (NH_4_OH) can be used as a neutralizing agent but poses the risk of chemical burns to fungal cells (Wang et al. 2014). The downstream processing of LA, including its separation, purification, and concentration, is another significant challenge in fungal fermentation. The recovery of LA from the fermentation broth is complicated by the presence of impurities, by-products, and residual biomass. Conventional purification methods, such as crystallization, distillation, and solvent extraction, are often energy-intensive and costly, generating considerable waste (Kim et al. 2022).

Although ion exchange and electrodialysis are more efficient alternatives, these methods still require further refinement (Boonkong et al. 2009). Developing more efficient, cost-effective, and environmentally friendly purification techniques remains a critical area of research. In addition to purification, scaling up fungal fermentation from the laboratory to industrial levels presents several challenges, such as maintaining consistent fermentation conditions in large bioreactors, ensuring uniform nutrient and oxygen distribution, and preventing contamination (Abdel-Rahman et al. 2013). The need for specialized equipment for pH control and temperature regulation further adds to the complexity and cost of industrial-scale operations.

## Techno-economic feasibility assessment of fermentation process

The techno-economic feasibility assessment of LA fermentation using *Rhizopus* species highlights both opportunities and challenges compared to conventional bacterial processes in terms of process's economic viability and operational considerations. While industrial LA production is predominantly performed by LAB under anaerobic conditions, fungal fermentation requires aerobic aeration, increasing energy consumption and thus raising operating costs (Perveen et al. 2023). Despite this, certain advantages in fungal systems, such as lower nutrient requirements and the ability to assimilate inorganic nitrogen, can help offset costs in other areas (Manandhar and Shah 2023). Liu et al. (2006) demonstrated that fed-batch culture of *Rhizopus sp.* MK-96-1196 achieved over 140 g/L of L-LA with an 83% yield in 96 h using a 3L air-lift reactor, with a productivity of 1.47 g/L/h (an initial glucose concentration of 60 g/L, using a 75% glucose solution to maintain 30 g/L glucose) (Liu et al. 2006). Comparative cost analyses have shown fed-batch fermentation to be the most cost-effective mode for LA production, with variable costs around 88% of batch processes and significantly lower than continuous systems (140%) (Liu et al. 2015). Despite its benefits, optimization strategies such as metabolic engineering and feedstock recycling are critical for enhancing the economic feasibility of fed-batch fermentation. LA prices vary based on application, ranging from $1.30/kg to $4.00/kg, depending on feedstock costs and market demands (Gezae Daful and Görgens 2017). However, direct cost comparisons between fungal and bacterial systems remain limited. Bacterial processes, typically operating without aeration, benefit from lower energy requirements, potentially making them more economically favorable despite higher organic nutrient demands. Techno-economic studies suggest that bacterial LA production costs, especially from pretreated lignocellulosic hydrolysates (pretreated corn stover hydrolysate), can reach approximately $0.56/kg under optimized (Biddy et al. 2016; Gezae Daful and Görgens 2017; Alves De Oliveira et al. 2018; Perveen et al. 2023). In fungal systems, higher capital investment for aeration equipment and operational energy costs would need to be balanced by process benefits, such as reduced nutrient costs and simultaneous purification steps (Brobbey et al. 2024). Furthermore, cellulase and enzyme costs remain significant for both systems, with ongoing improvements in enzyme efficiency being critical.

Life cycle assessments (LCA) and techno-economic analyses are crucial for understanding the environmental and economic sustainability of LA production. These assessments cover all stages from raw material acquisition to final disposal, highlighting critical factors such as fossil energy use, GHGs emissions, and water consumption. The price of LA varies depending on its application—food, pharmaceuticals, and PLA production. For PLA synthesis, the optical purity of LA is a critical parameter, as the mechanical, thermal, and biodegradability properties of PLA are highly influenced by the ratio of L- and D-lactic acid isomers. High optical purity, particularly of L-LA, is required to produce high-quality, high-molecular-weight PLA (Tashiro et al. 2013). Fungal fermentation, particularly using *Rhizopus* species, typically produces L(+)-lactic acid with high optical purity, often exceeding 99% under optimized conditions, making it suitable for PLA manufacturing (Liaud et al. 2015; Trakarnpaiboon et al. 2017). In addition to this advantage LCA demonstrate that PLA derived from LA has a lower carbon footprint than petrochemical-based polymers. PLA production consumes 25% to 55% less fossil energy compared to petrochemical-based plastics, significantly reducing GHG emissions (Ahmad et al. 2020). Moreover, PLA biodegradation releases CO_2_ equivalent to that absorbed by plant-based feedstocks during growth, establishing a carbon–neutral profile (Ilyas et al. 2022).

Overall, while LA fermentation using *Rhizopus* species offers environmental advantages and the potential for lower nutrient costs, its higher energy demands due to aeration present a key economic challenge. A thorough, direct techno-economic comparison with established bacterial processes- considering both operating and investment costs, is necessary to clearly establish its industrial viability.

## Conclusion and future perspectives

The increasing demand for LA in industries like food, chemicals, pharmaceuticals, biomedical, cosmetics, and biodegradable plastics (PLA) highlights the need for efficient and cost-effective fermentation processes. Biological methods are preferred over chemical synthesis for producing stereospecific LA, with *Rhizopus* sp. offering advantages such as amylolytic activity, low nutrient requirements, and simpler downstream processing. *Rhizopus* strains can ferment various renewable substrates, including lignocellulosic materials and agricultural waste, providing cost-effective alternatives to edible starch. Optimal LA production relies on conditions such as temperature (30–35 °C), pH (5.5–6.0), and substrate concentration, with *R. oryzae* identified as an effective microorganism for high yields. While batch fermentation is commonly used due to low costs, fed-batch and continuous methods can further enhance yields. LA purification, a significant cost factor, employs methods like precipitation, distillation, and membrane separation, with electrodialysis emerging as an efficient and eco-friendly option.

Despite these advantages, commercial scale LA production using *Rhizopus* faces several challenges. High energy consumption due to aerobic conditions increases operational costs, and downstream purification remains a significant cost driver. Substrate costs and availability, product inhibition, and variability in strain performance further complicate large-scale operations. Additionally, limited techno-economic and life-cycle data for fungal systems hinder direct comparisons with established bacterial processes, making industrial feasibility assessments more complex.

To enhance industrial viability, future research should focus on developing improved *Rhizopus* strains with higher substrate tolerance and productivity, employing low-cost renewable feedstocks such as agro-industrial residues, and optimizing aeration and process parameters to reduce energy use. Integration of enzyme recycling or co-culture systems can improve substrate conversion, while advanced, energy-efficient purification technologies should be applied to lower costs and environmental impact. Comprehensive techno-economic and life cycle assessments, along with continued advancements in fermentation and purification technologies are crucial to guide sustainable scale-up and ensure the long-term success of commercial LA production.

## Data Availability

The data presented in Tables 1, 3, and 5 were specifically prepared for this review. No other new data were created or analyzed in this study.
